# Inhibition of Phagocytosis by Silibinin in Mouse Macrophages

**DOI:** 10.3390/cimb45100513

**Published:** 2023-10-06

**Authors:** Kyung-Hoon Sun, Min-Young Lee, Young-Jin Jeon

**Affiliations:** 1 Department of Emergency Medicine, College of Medicine, Chosun University, Gwangju 61452, Republic of Korea; skhkorea@chosun.ac.kr; 2 Department of Pharmacology, College of Medicine, Chosun University, Gwangju 61452, Republic of Korea; minyoung0209@hanmail.net

**Keywords:** macrophages, phagocytosis, silibinin, lamellipodia, filopodia

## Abstract

This study investigated the effects of silibinin, derived from milk thistle (*Silybum marianum*), on lipopolysaccharide (LPS)-induced morphological changes in mouse macrophages. Silibinin was treated at various doses and time points to assess its effects on macrophage activation, including morphological changes and phagocytosis. Silibinin effectively inhibited LPS-induced pseudopodia formation and size increase, while unstimulated cells remained round. Silibinin’s impact on phagocytosis was dose- and time-dependent, showing a decrease. We explored its mechanism of action on kinases using a MAPK array. Among the three MAPK family members tested, silibinin had a limited effect on JNK and p38 but significantly inhibited ERK1/2 and related RSK1/2. Silibinin also inhibited MKK6, AKT3, MSK2, p70S6K, and GSK-3β. These findings highlight silibinin’s potent inhibitory effects on phagocytosis and morphological changes in macrophages. We suggest its potential as an anti-inflammatory agent due to its ability to target key inflammatory pathways involving ERK1/2 and related kinases. Overall, this study demonstrates the promising therapeutic properties of silibinin in modulating macrophage function and inflammation.

## 1. Introduction

Silibinin, a prominent constituent of silymarin, represents a key bioactive compound derived from the seeds of the milk thistle plant (*Silybum marianum*) [1]. With a history deeply rooted in traditional medicine, it has been harnessed for ages in the treatment of various liver disorders. Robust scientific inquiry has unveiled the impressive hepatoprotective potential of silymarin, with silibinin at its core, countering the detrimental effects of an array of chemical agents, including microcystin, ochratoxin, ethanol, phenylhydrazine, and acetaminophen [2,3,4,5]. Beyond its remarkable hepatoprotective attributes, silibinin exhibits a diverse spectrum of biological activities. These encompass anti-inflammatory properties and its compelling role as an anti-carcinogenic agent, further expanding its potential therapeutic utility [6,7,8,9]. Such multifaceted characteristics underscore the significance of silibinin in both traditional medicine and modern pharmacology.

Macrophages, recognized as one of the most versatile cell types within the immune system, fulfill indispensable roles in maintaining overall physiological equilibrium. Their paramount function centers around phagocytosis, an intricate process through which they engulf and digest a diverse array of entities, encompassing microbial invaders, detritus from deceased cells, and even cancerous cells [10]. However, the multifaceted contributions of macrophages extend far beyond phagocytosis. Macrophages actively participate in the orchestration of immune responses by recruiting other lymphocytes and finely modulating adaptive immunity, guided by environmental cues. This dynamic behavior leads to a process of polarization and differentiation, giving rise to two distinct phenotypes: M1 and M2 macrophages, each characterized by its unique functional profile [11]. M1 macrophages emerge as pivotal sentinels in the arena of host defense, staunchly combating viral, bacterial, and tumorous threats. In contrast, M2-polarized macrophages, often referred to as activated macrophages, redirect their efforts towards tissue repair and the resolution of inflammatory processes. Notably, a close relative of M2 macrophages, known as tumor-associated macrophages (TAMs), assumes a prominent role in shaping the tumor microenvironment. Their intricate interplay involves the regulation of factors that significantly influence oncogenesis, thus underscoring the profound impact of macrophages on diverse physiological and pathological processes [12,13].

M1 macrophages, one of the pivotal cellular components of the immune system, respond to a cadre of stimulating factors that encompass lipopolysaccharide (LPS), interferon-gamma (IFN-γ), and granulocyte–macrophage colony-stimulating factor (GM-CSF) [14]. In particular, LPS, through its interaction with toll-like receptor 4 (TLR4), assumes a central role in inciting classical macrophage activation, thereby orchestrating the upregulation of genes integral to cytokine receptors, cell adhesion molecules, and activation markers. Simultaneously, IFN-γ, predominantly secreted by Th1 lymphocytes, serves as a potent stimulator of macrophages, and GM-CSF operates as an additional promoter of M1 polarization [14]. M1 macrophages, thus activated, unleash a torrent of pro-inflammatory cytokines and unleash a slew of toxic substances, including nitric oxide (NO) and reactive oxygen intermediates. These formidable armaments are strategically employed in the service of host defense against invading pathogens and malignant tumors [14,15]. Nevertheless, it is essential to acknowledge that while M1 macrophages play a crucial role in immune responses, their unrestrained activation can potentially precipitate inflammatory disorders, thereby exerting adverse consequences on host health.

The activation of macrophages induced by LPS has a profound impact on their morphology, giving rise to a spectrum of alterations [16]. These changes encompass an increase in cell size, the emergence of lamellipodia and filopodia, and significant modifications in cell adhesion, migration, and phagocytosis processes [16]. Lamellipodia, distinguished by their resemblance to thin sheets and their location at the leading cellular edge, are particularly rich in the cytoskeletal protein actin. In contrast, filopodia are slender, actin-laden protrusions that extend beyond the confines of lamellipodia [17]. These intricate structures are of paramount importance for the phagocytic prowess of macrophages. In the realm of macrophage physiology, lamellipodia perform a dual role: they serve as both the engine propelling cell migration and as essential participants in the process of phagocytosis. In migration, lamellipodia function akin to a motor, exerting forces that pull the cell forward, facilitating its movement [18,19,20]. On the other hand, filopodia play a pivotal role in phagocytosis by attaching to objects of interest and exerting pulling forces to draw these objects closer to the cell, thereby aiding in their engulfment [18,19,20]. This orchestration of cytoskeletal dynamics and adhesion processes, integral to cellular migration, is tightly regulated by Rho GTPases. These regulatory molecules, which encompass RhoA, a member of the Rac subfamily, and Cdc42, play pivotal roles in governing the intricate dance of the cell during migration [21]. Further complicating this landscape of cellular dynamics is the influence of the nuclear transcription factor NF-κB. It wields a significant role in modulating actin filament dynamics through integrin-mediated signaling pathways. Additionally, NF-κB exerts control over morphological transformations within the cell, including the formation of lamellipodia [22].

In the current study, we investigated the effects of silibinin on phagocytosis function of macrophages and morphological changes, including lamellipodia and filopodia, which are crucial for the phagocytic function of macrophages. These structures facilitate the movement of macrophages towards bacteria and the attachment and pulling of bound objects.

## 2. Materials and Methods

### 2.1. Materials

The following materials were purchased for the study: silibinin and LPS (*Salmonella typhosa*) from Sigma (St. Louis, MO, USA); the anti-F-actin antibody from Santa Cruz Biotechnology (Santa Cruz, CA, USA); a human phospho-MAPK array kit from R&D Systems (Minneapolis, MN, USA); and pHrodo^®^ green *Escherichia coli* bioparticles from Essen BioScience (Ann Arbor, MI, USA).

### 2.2. Cell Culture and Adhesion Assay

RAW 264.7 cells were obtained from the American Type Culture Collection (Bethesda, MD, USA) and cultured in a 5% CO_2_ environment at 37 °C. Macrophages were cultured in DMEM supplemented with 10% FBS, 2 mM L-glutamine, and penicillin-streptomycin. Cells were treated with silibinin (50 μg/mL) in the presence of LPS (200 ng/mL) for 18 h. Cells were collected and re-plated at a density of 1 × 10^5^ cells/mL. After 30 min, unattached cells were removed by washing with phosphate-buffered saline (PBS) 3 times. Cell adhesion was calculated by counting the attached cells and expressing this number as a percentage of the total cells. Cytotoxicity was assessed using the 3-(4,5-dimethylthiazol-2-yl)2,5-diphenyl tetrazolium bromide (MTT) cleavage assay conducted with an Elx800 microplate reader. Cells were exposed to silibinin and/or LPS for 18 h. The MTT reagent was added directly to the cell culture medium. Following a 4 h incubation, the medium containing MTT was removed, and the formazan crystals were solubilized with dimethyl sulfoxide. The absorbance was measured at 570 nm. The MTT assay results indicated that the viability of all treated cells exceeded 80%.

### 2.3. Immunofluorescence Staining

Macrophages were exposed to LPS on cover slides. The cells were washed with PBS three times, fixed with 4% paraformaldehyde for 10 min at 25 °C, and rinsed again with PBS. Subsequently, the cells were blocked with 1% bovine serum albumin and incubated with the primary antibody overnight. After the incubation, the cells were washed with TBS and incubated with fluorescein isothiocyanate-conjugated IgG for 1 h. Following a rinse, the cells were mounted and observed at 488 nm using a confocal microscope (FV300; Olympus, Tokyo, Japan).

### 2.4. Scanning Electron Microscopy

Cover slides containing macrophages were subjected to culture with silibinin and/or LPS for varying durations of 0.5, 1.5, 3, 6, 12, or 24 h. Afterward, the plates were washed three times with PBS and left to air-dry at 25 °C. The cells were then fixed with 2% osmium tetroxide in PBS (2 mL per well) for 4 h and washed three times with PBS. To facilitate dehydration, the cells were immersed in ethanol of increasing concentrations (40, 50, 60, 70, 80, 90, or 100%) for 10 min. Following this, the slides were air-dried and coated with an E-1030 ion sputtering coating machine (Hitachi High-Technologies, Tokyo, Japan). Finally, an S-4800 field emission scanning electron microscope (Hitachi High-Technologies) was utilized to observe the slides for a duration of 30 min.

### 2.5. Phagocytosis Assay

Macrophages were exposed to silibinin and/or LPS for 24 h in 96-well plates. The phagocytosis activities were assessed using the IncuCyte^®^ ZOOM live-cell imaging system (software 2015A). Time-lapse movies were captured using the IncuCyte^®^ ZOOM, enabling real-time visualization of mouse macrophage cells engulfing pHrodo green *E. coli* bioparticles. The fluorescence of the phagosome, which indicates the acidic environment, increased. The integrated image analysis tools of IncuCyte^®^ ZOOM were employed to detect and quantify the green fluorescent signals throughout the entire duration of the assay.

### 2.6. MAPKs Array

Macrophages were exposed to silibinin for 1 h and treated with LPS for 20 min. Cell lysates were then prepared and analyzed the MAPK phosphorylation using human phsoho-MAPK array kit. Cell lysates were diluted, mixed with a cocktail of biotinylated detection antibodies, and incubated overnight with the nitrocellulose membranes spotted capture and control antibodies. The membranes were then washed and detected the chimiluminescence. The intensity of the dots was quantified by densitometric analysis.

### 2.7. Statistical Analysis

The experiments were conducted quantitatively, and each experiment was independently repeated at least three times. The data presented in the results represent the mean value along with the standard deviation (SD) for each experimental group. Unless stated otherwise in the figures, a *p*-value of less than 0.05 was considered significant. Statistical analyses were performed using the Student’s *t*-test.

## 3. Results

### 3.1. Inhibition of Macrophage Activation by Silibinin in LPS-Stimulated RAW264.7 Cells

**Figure 1:**
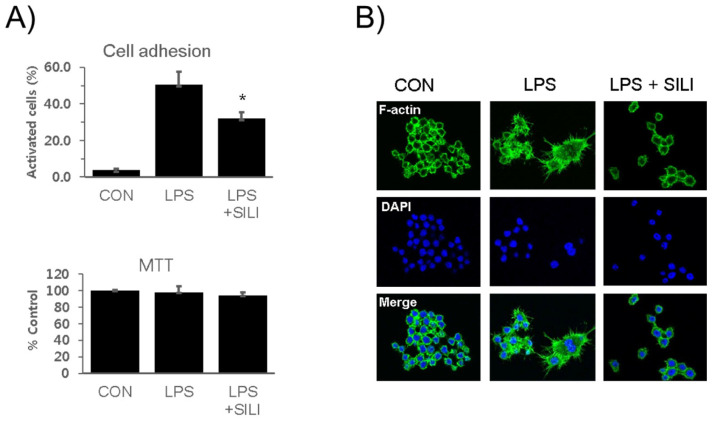
Silibinin inhibited macrophage activation in LPS-stimulated macrophages. (**A**) RAW264.7 cells were treated with silibinin (50 μg/mL) in the presence of LPS for 18 h. Cells were harvested, washed, plated in 6-well plates (5 × 10^5^/mL) for 30 min, washed, and analyzed for adhesion using microscopy. Attached cells were counted before and after washing. Each column shows the mean ± SD of triplicate measurements. * *p* < 0.05 compared with the control, as determined by Student’s *t*-test. (**B**) The cells were treated with silibinin (50 μg g/mL) in the presence of LPS for 18 h on cover slides and then subjected to immunofluorescence staining for F-actin and DAPI staining.

RAW 264.7 cells were cultured in the presence of silibinin and LPS for 18 h. The cells were collected and analyzed the adhesion activity. Cell attachment activities were strongly increased by LPS stimulation, whereas LPS-induced cell adhesion activity was significantly inhibited by silibinin (Figure 1A). MTT assay showed that no cytotoxic effects of the silibinin were observed. Immunofluorescence staining of the cells further confirmed that silibinin inhibited the morphological changes induced by LPS (Figure 1B).

**Figure 2:**
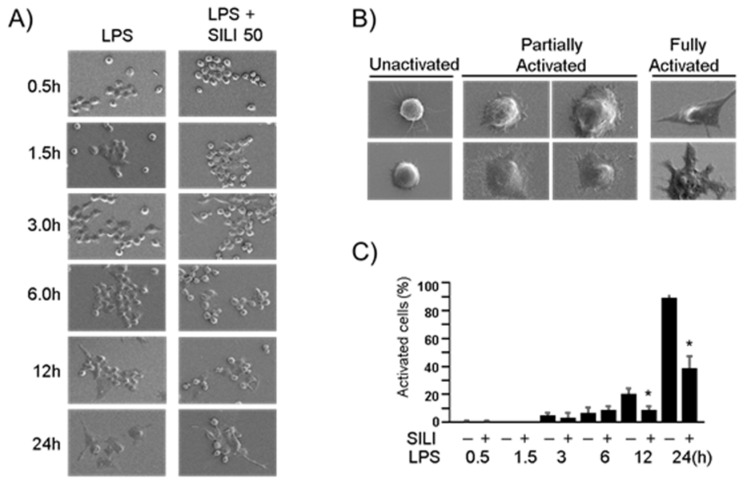
Silibinin inhibited morphological changes in LPS-stimulated macrophages. (**A**) RAW264.7 cells were treated with silibinin (50 μg/mL) in the presence of LPS for 0.5, 1.5, 3, 6, 12, or 24 h on cover slides. The cells were then subjected to scanning electron microscopy. (**B**) Representative photographs of macrophages. (**C**) Fully activated cells were counted, and the results are expressed as a percentage of the total number of cells. Each column shows the mean ± SD of triplicate measurements. * *p* < 0.05 compared with the control, as determined by Student’s *t*-test.

To further examine the morphological changes, scanning electron microscopy was employed. In the presence of LPS, macrophages were treated with silibinin for varying durations of 0.5, 1.5, 3, 6, 12, or 24 h. Interestingly, some cells treated with silibinin maintained their inactivated round sphere shape (Figure 2A). Partially activated cells displayed attachment and spreading of lamellipodia while maintaining a round shape. Fully activated macrophages exhibited an irregular shape with more extensively extended lamellipodia (Figure 2B). The quantification of fully activated cells indicated that silibinin treatment inhibited LPS-induced morphological changes in macrophages (Figure 2C). These findings provide compelling evidence of the potent role of silibinin in curtailing morphological alterations in mouse macrophages, shedding light on its regulatory impact in this context.

### 3.2. Inhibition of Phagocytosis by Silibinin in LPS-Stimulated Macrophages 

**Figure 3:**
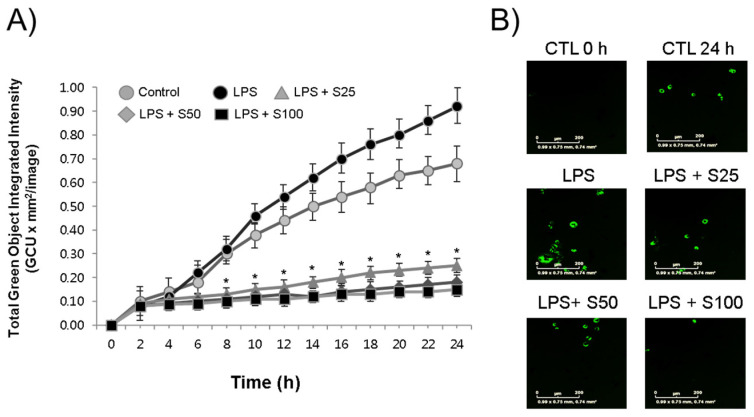
Silibinin inhibited phagocytosis in LPS-stimulated macrophages. (**A**) RAW264.7 cells were treated with silibinin (25, 50, or 100 μg/mL) in the presence of LPS for 24 h in 96-well plates. The cells were then subjected to phagocytosis assay. (**B**) Representative photographs of macrophages with fluorescence. Each time point shows the mean ± SD of triplicate experiments. * *p* < 0.05 compared with the control, as determined by Student’s *t*-test.

To assess the impact of silibinin on macrophage function, RAW264.7 cells were treated with silibinin in the presence of LPS, and their phagocytic activity was examined using the IncuCyte^®^ ZOOM live-cell imaging system. The results revealed that LPS stimulation led to an increase in macrophage engulfment of *E. coli* bioparticles, accompanied by a time-dependent rise in green fluorescence (Figure 3A). Interestingly, it was observed that even the LPS-untreated control macrophages exhibited heightened phagocytosis activities. This phenomenon could potentially be attributed to the inherent stimulatory effects of the *E. coli* bioparticles themselves on the macrophages. Significantly, when subjected to silibinin treatment, the phagocytic activities of the macrophages were distinctly hindered. This inhibition was evident in the form of weaker fluorescence compared to the control cells, as vividly depicted in the representative photographs showcased in Figure 3B.

**Figure 4:**
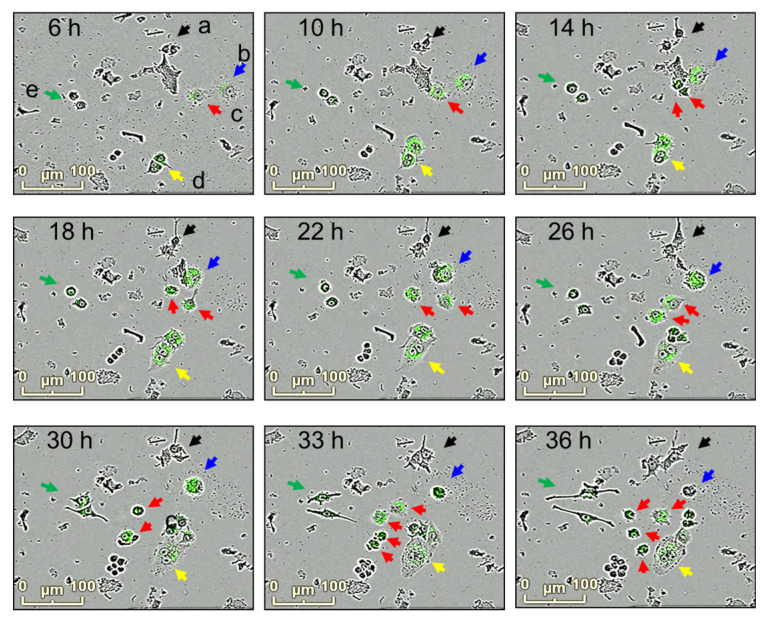
Morphological changes and phagocytosis activity in macrophage cultures. RAW264.7 cells were incubated with *E. coli* bioparticles for 36 h in 96-well plates. Morphological changes and phagocytosis activities were analyzed using the IncuCyte^®^ ZOOM live-cell imaging system. Snapshot of bright-field and florescence photographs were taken at 6, 10, 14, 18, 22, 26, 30, 33, and 36 h. Macrophages with changes in morphology and phagocytosis activities are shown (a; black, b; blue, c; red, d; yellow, e; green).

The relationship between morphological changes and phagocytosis activities in macrophages was further investigated. RAW264.7 cells were exposed to *E. coli* bioparticles, and their fluorescence and morphological changes were examined using merged images of bright-fields and fluorescence (Figure 4). Several observations were made: (a) some macrophages remained inactive in phagocytosis (labeled as “a”); (b) other macrophages actively engulfed the bioparticles, resulting in an enlargement of their size, and eventually underwent cell death; (c) some macrophages displayed active phagocytosis, exhibited movement, growth, and division; (d) macrophages that engaged in phagocytosis but did not divide became multinucleated giant cells with cytoplasmic projections on their cellular surface; (e) certain macrophages showed elongated cell bodies with cytoplasmic projections at the apical ends but had low phagocytosis activity. Macrophages demonstrating high phagocytosis activities (b and c) were identified as M1-polarized macrophages, whereas those with low phagocytosis activities (d and e) were classified as M2-polarized macrophages. To comprehensively investigate the effects of silibinin on macrophage differentiation, it would be beneficial to quantify both M1- and M2-polarized cells and assess markers associated with these polarization states.

### 3.3. Inhibition of MAPKs by Silibinin in LPS-Stimulated RAW264.7 Cells

**Figure 5:**
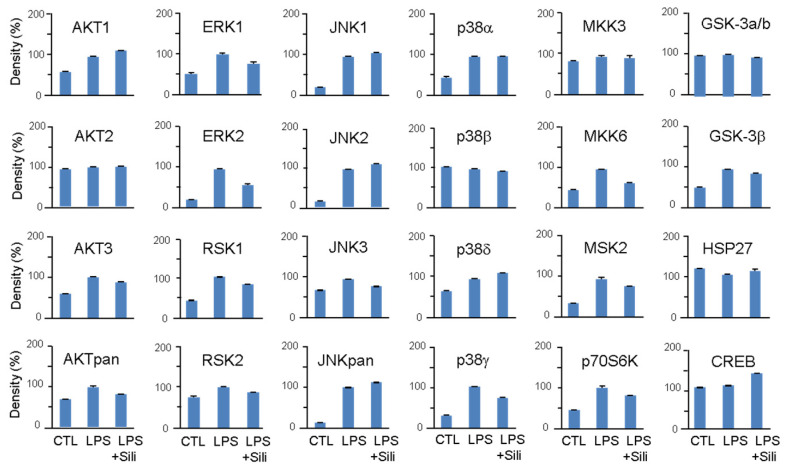
Effects of silibinin on LPS-induced MAPK activation. RAW264.7 cells were pretreated with silibinin (50 μg/mL) for 1 h and then treated with LPS for 20 min. Cell lysates were prepared and used for MAPK phosphorylation analysis using a MAPK array kit. The intensity of the dots was quantified by densitometric analysis.

The effects of silibinin on the MAPK signaling pathway were investigated to understand its mechanism of action. Macrophage cells were treated with silibinin for 1 h, followed by LPS treatment for 20 min. The phosphorylation of various MAPK proteins was analyzed using a MAPK array kit in cell lysates (Figure 5). While some antibodies indicated cross-reactivity with mouse phospho-MAPK, and LPS stimulation notably induced phosphorylation, it is important to acknowledge the potential for nonspecific antibody detection. Therefore, we primarily analyzed the MAPK array results in terms of their broader impact on MAPK pathways rather than focusing solely on specific kinases.

LPS stimulation significantly increased the phosphorylation of AKT, ERK, RSK, JNK, p38, MKK6, MSK2, p70S6K, and GSK-3β. Silibinin did not inhibit the phosphorylation of JNK and p38, but it significantly inhibited the phosphorylation of ERK1/2 (Figure 5). Additionally, silibinin decreased the phosphorylation levels of RSK1/2, MKK6, MSK2, and p70S6K, which were initially increased by LPS stimulation.

These findings indicate that silibinin acts on the MAPK pathway by selectively inhibiting the phosphorylation of ERK1/2 and modulating the phosphorylation of downstream targets, including RSK1/2, MKK6, MSK2, and p70S6K.

## 4. Discussion

Silibinin, a polyphenolic flavonoid and a major component of milk thistle extract, was found to have strong anti-inflammatory properties, as demonstrated in this study. The research showed that silibinin effectively inhibited phagocytosis, a key inflammatory function of macrophages responsible for engulfing and digesting foreign and endogenous substances to protect the host from pathogens and cancer cells [10]. The study utilized *E. coli* bioparticles to assess phagocytosis, and the results indicated that silibinin dose- and time-dependently inhibited this process (Figure 3A). Additionally, the study used a pH-sensitive dye-conjugated *E. coli* assay to observe phagocytosis in LPS-activated macrophages, where the dye emitted green fluorescence in acidic phagosomes (Figure 3B). Notably, even in the absence of LPS stimulation, macrophages displayed significant phagocytic activity, likely due to the presence of LPS in the bacteria, which activated macrophages through membrane receptors such as TLR4. TLR4 is a pattern recognition receptor (PRR) found on macrophages and other innate immune cells, recognizing pathogen-associated molecular patterns (PAMPs) from microorganisms. Activation of TLRs initiates inflammatory responses [23]. TLR4, in particular, recognizes LPS, while other TLRs recognize different components of bacteria, such as lipopeptides, peptide glycans, or nucleic acids, thus activating downstream signaling pathways and transcription factors such as NF-κB, leading to the expression of inflammatory cytokine genes [24].

Another observation made in this study was the inhibitory effect of silibinin on morphological changes in LPS-stimulated macrophages, specifically the formation of lamellipodia and filopodia (Figure 2). Scanning electron microscopy revealed that LPS induced changes in macrophage morphology, including increased cell size and the formation of lamellipodia and filopodia. Lamellipodia are thin, actin-rich protrusions at the leading edge of the cell, while filopodia are spike-like extensions extending beyond lamellipodia [17]. Since both lamellipodia and filopodia are essential for macrophage phagocytic function, the inhibition of these morphological changes by silibinin correlated well with the observed inhibition of phagocytosis. During phagocytosis, lamellipodia and filopodia aid in cell movement towards bacteria and facilitate attachment and pulling of the bound material towards the cell [18,19,20]. The inhibitory effects of silibinin on phagocytosis and macrophage morphology provide further evidence of its potential as an anti-inflammatory agent. These findings align with previous studies that demonstrated the anti-inflammatory effects of silibinin in models of endotoxin-induced uveitis and lung injury [25,26]. Silibinin treatment in rats significantly reduced inflammatory cell infiltration in the eyes and inhibited the production of inflammatory markers, such as protein, NO, PGE2, iNOS, and COX-2. Silibinin also suppressed the recruitment of airway inflammatory cells, including macrophages, in a lung injury model. Moreover, silibinin has been shown to decrease the production of inflammatory cytokines, including IL-1β and TNF-α [27]. These effects on macrophage function and morphology, along with the attenuation of inflammatory responses, support the potential of silibinin as an anti-inflammatory agent.

To explore the underlying mechanism of silibinin’s inhibitory effect on macrophage activation, the study investigated its impact on kinases using a MAPK array. Upon LPS stimulation, macrophages activate MAPK family members, including ERK1/2, p38, and JNK, through phosphorylation [28]. The study revealed that silibinin significantly inhibited the phosphorylation of ERK1/2 and its downstream target RSK1/2. However, the effects of silibinin on p38 and JNK were not conclusive; it inhibited p38γ and JNK3 but not p38α, p38β, p38δ, JNK1, JNK2, and JNKpan. Silibinin also inhibited other kinases such as MKK6, AKT3, MSK2, p70S6K, and GSK-3β. Previous studies have shown that silibinin can prevent the activation of MAPKs and NF-κB in different cell types and signaling pathways, such as osteoclast precursor cells, thyroid and breast cancer cells, and gastric cancer cells [29,30,31,32]. Silibinin inhibited receptor activator of NF-κB ligand (RANKL)-induced osteoclastogenesis from RAW264.7 cells, as well as from bone marrow-derived monocyte/macrophage cells [29]. Although silibinin’s inhibition of MAPK phosphorylation has been observed in various studies, the exact direct targets of silibinin in these pathways have yet to be identified. Further analysis of MAPK phosphorylation is required to investigate the mechanism of silibinin on phagocytosis inhibition.

**Figure 6:**
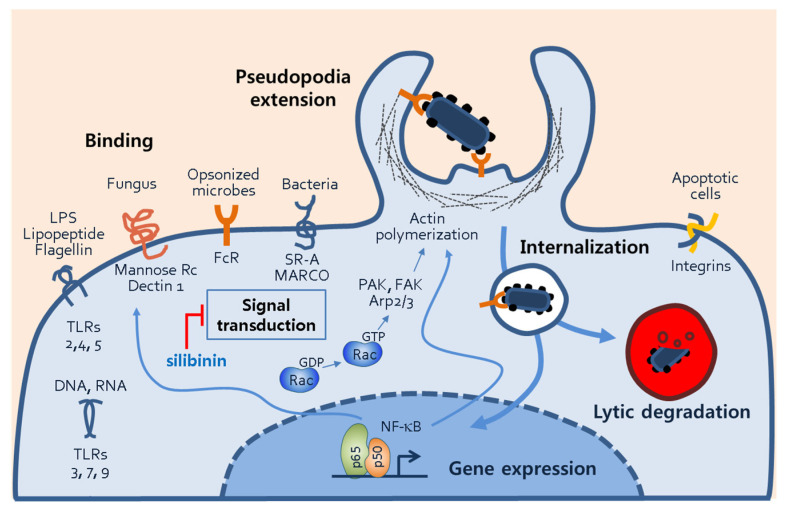
Phagocytic mechanisms of macrophages. The figure illustrates several processes of macrophage phagocytosis, including phagocytosis receptor–ligand binding, signal transduction, pseudopodia extension, internalization of microbes, and phagosomal degradation. Through the phagosomal processes, transcription factors such NF-κB are activated, and they subsequently induce the gene expression of proteins involved in phagocytosis. Abbreviations: TLR, toll-like receptor; FcR, fragment crystallizable receptor; SR-A, scavenger receptor-A, NF-κB, nuclear factor of kappa light chain; PAK, p21-activated kinase; FAK, focal adhesion kinase; Arp2/3, actin-related proteins 2/3.

An important finding of this study was the inhibition of phagocytosis by silibinin. This inhibition may be attributed to the prevention of morphological changes, particularly the formation of lamellipodia and filopodia. During phagocytosis, cellular receptors bind to bacterial surface antigens, facilitated by unique molecular patterns, opsonins, and apoptotic cells (Figure 6). Macrophages possess several PRRs that specifically bind to PAMPs. Examples include the mannose receptor and Dectin-1, which bind to fungi with surface polysaccharides [33,34]. Scavenger receptors like SR-A and MARCO recognize surface molecules on both Gram-negative and Gram-positive bacteria [35,36,37]. Fragment crystallizable receptors on phagocytes recognize opsonins such as immunoglobulin G (IgG), C3b, or iC3b bound to foreign materials [38,39]. Phagocytosis of apoptotic cells is also vital for cell turnover in the body. Although the study demonstrated the inhibition of macrophage activation by silibinin, it remains unclear whether silibinin directly blocks receptor–ligand interactions.

Silibinin’s inhibition of phagocytosis and macrophage activation may involve the inflammatory transcription factor NF-κB. NF-κB is activated by cellular receptors and ligand binding and plays a crucial role in phagocytosis by regulating gene expression (Figure 6). NF-κB is known to regulate actin cytoskeleton dynamics and induce morphological changes such as lamellipodia formation [22]. Additionally, NF-κB drives the expression of inflammatory mediators including iNOS, COX-2, and cytokines [40]. Cell adhesion molecules such as VCAM-1, ICAM-1, and E-selectin may also be targeted by NF-κB to mediate macrophage morphological changes [41,42,43].

The remarkable capacity of silibinin to inhibit the production of ROS and NO is indeed noteworthy. This inhibition, in turn, acts as a barrier to the activation of NF-κB, a pivotal player in the macrophage activation process triggered by oxidative stress. Silibinin, known for its potent antioxidant properties, has been substantiated in prior research as a formidable inhibitor of ROS and NO production [27,44]. Given the critical role of these pathways in inflammation, silibinin holds promise as a potential anti-inflammatory agent. These findings underscore the far-reaching implications of silibinin in the context of inflammation and its therapeutic promise.

One limitation of our study is the reliance on a single mouse cell line, RAW264.7 cells. To strengthen the evidence supporting silibinin’s effects, further investigations should encompass a broader spectrum of cell types, including human monocyte cell lines such as THP-1, primary human peripheral blood mononuclear cells (PBMCs), and murine bone marrow-derived macrophages (BMDMs). Human cell lines and primary cells yield results that hold more direct relevance to human biology. This becomes particularly critical when examining diseases or conditions characterized by substantial disparities between human and mouse physiology. The use of THP-1 cells, PBMCs, and BMDMs enables a more precise exploration of human-specific inflammatory responses, which may not be faithfully recapitulated in mouse cell lines. Such an approach is indispensable for gaining insights into the intricate role of inflammation in various diseases. In summary, while mouse cell lines, such as RAW264.7, undeniably serve their purpose in research, the inclusion of human cell lines and primary cells enriches our investigations, providing a more comprehensive and clinically pertinent perspective. This becomes especially valuable when delving into the realms of human diseases, immunology, drug responses, and the complexities of inflammation.

In summary, the present study demonstrated that silibinin, a polyphenolic flavonoid derived from milk thistle, possesses potent anti-inflammatory properties. Silibinin inhibited phagocytosis and morphological changes in LPS-stimulated macrophages. The inhibition of phagocytosis by silibinin correlated with its ability to prevent the formation of lamellipodia and filopodia, which are essential for macrophage function. Silibinin also showed inhibitory effects on various kinases, particularly ERK1/2, and downstream signaling molecules. The precise direct targets of silibinin in these pathways require further investigation. Moreover, silibinin’s inhibition of phagocytosis and macrophage activation may involve the modulation of receptor–ligand interactions and the inflammatory transcription factor NF-κB. Silibinin’s antioxidative properties, by inhibiting ROS and NO production, further contribute to its anti-inflammatory effects. Overall, these findings highlight the potential of silibinin as a promising anti-inflammatory agent, but additional research is needed in order to fully elucidate its molecular mechanisms of action.

## Data Availability

The data presented in this study are available in this article.
